# Optic Nerve Sheath Measurements for Noninvasive Monitoring of Malignant Supratentorial Infarcts: A Systematic Review and Meta-Analysis

**DOI:** 10.7759/cureus.95918

**Published:** 2025-11-01

**Authors:** Ed Sherman T Chu

**Affiliations:** 1 Neurological Surgery, Davao Doctors Hospital, Davao, PHL

**Keywords:** diagnostic utility, malignant supratentorial infarcts, noninvasive monitoring, optic nerve sheath diameter, stroke

## Abstract

Introduction: Early detection of malignant supratentorial infarcts, particularly malignant middle cerebral artery (MCA), is crucial; however, advanced imaging techniques such as MRI and PET are often inaccessible in resource-limited areas. A potential solution for early risk stratification of stroke patients is the optic nerve sheath diameter (ONSD) measured by transorbital ultrasound. The purpose of this study was to evaluate the utility of optic nerve sheath imaging measurements in recognizing malignant infarcts. A meta-analysis on ONSD measurement has been done for acute ischemic stroke patients.

Methods: Following Preferred Reporting Items for Systematic Reviews and Meta-Analyses (PRISMA) 2022 guidelines, a comprehensive search was conducted for studies published between January 2000 and December 2023. A total of six eligible studies were included. These studies were observational studies involving adult patients diagnosed with malignant supratentorial infarcts who underwent ONSD assessment using ultrasonography, computed tomography (CT), or magnetic resonance imaging (MRI). Data extraction and risk of bias assessments were performed independently. A meta-analysis was conducted to pool estimates of mean ONSD measurement, sensitivity, specificity, positive predictive value (PPV), negative predictive value (NPV), and accuracy using the metamean() and metaprop() functions from the meta R package.

Results: Four studies (n = 170) show a high detection rate of 89.01% (95% CI: 82.88-93.13) with low heterogeneity (I² = 21.3%) based on CT findings of malignant middle cerebral artery (MCA) infarction or progression of acute ischemic stroke. Six studies (n = 314) reporting ONSD showed a pooled mean of 5.50 mm (95% CI: 5.24-5.76 mm), though heterogeneity was substantial (I² = 97.0%). Findings show a sensitivity of 89.01%, specificity of 82.94% to 86.63%, PPV of 81.37%, NPV of 92.57%, and overall diagnostic accuracy of 87.82%. An optimal ONSD cutoff value across studies of 4.70 mm to 5.60 mm, with 5.60 mm, demonstrates the highest diagnostic yield (Youden’s Index = 0.8947).

Conclusion: ONSD measurements can be used in determining malignant infarcts.

## Introduction and background

Stroke remains a critical global health issue, particularly in low- and middle-income countries where resources are limited. The burden of stroke is expected to rise dramatically, with projections indicating an 81% increase in incidents and a 71% increase in prevalent cases from 2021 to 2050. Public health initiatives must prioritize interventions that address the diverse needs of various demographic groups to reduce stroke risk and mitigate disparities [1]. As stroke research advances, novel diagnostic tools and interventions are emerging, offering hope for improved patient outcomes. Among these, optic nerve sheath diameter (ONSD) measurement has gained attention as a noninvasive method for monitoring intracranial pressure (ICP) in critically ill neurological patients, including those with stroke.

Stroke is a dynamic condition that ranges from transient ischemic attacks (TIAs) to full-blown brain infarction. Malignant middle cerebral artery (MCA) infarction, a severe form of stroke, involves more than 50% of the MCA territory and is characterized by life-threatening complications such as space-occupying brain edema, midline shift, and herniation. Without surgical intervention, fatality rates can reach 80%, and survivors often face permanent disabilities. Advanced imaging techniques like magnetic resonance imaging (MRI) and positron emission tomography (PET) provide better predictions but were often inaccessible, especially in resource-limited settings or for severely ill patients [2].

ONSD measurement has emerged as a promising, noninvasive alternative for ICP monitoring. Studies have demonstrated its accuracy in detecting elevated ICP, making it a valuable tool in neurocritical care [3-4]. However, its application in stroke management, particularly in predicting malignant progression, remains underexplored. Recent research highlights the potential of ONSD in identifying patients at high risk for malignant infarction, offering a cost-effective and feasible solution for settings with limited resources [5-6]. For instance, Lee et al. found that ONSD measurements correlated strongly with ICP changes in stroke patients, suggesting its utility in early detection of malignant edema [5]. Similarly, Li et al. reported that ONSD could predict clinical deterioration in ischemic stroke patients, providing a window for timely intervention [6].

Despite these promising findings, uncertainties remain. The optimal cutoff values for ONSD in stroke patients, their reliability across different populations, and their integration into existing stroke care protocols require further investigation. Additionally, the variability in measurement techniques and interobserver reliability poses challenges to its widespread adoption [7]. This systematic review and meta-analysis aim to consolidate the latest evidence on ONSD measurements in ischemic stroke management, addressing these gaps and providing a comprehensive evaluation of its diagnostic and prognostic utility. Previous systematic review and meta-analysis have focused on the utility of ONSD in increased ICP cases in traumatic brain injury, but no studies have focused on acute ischemic stroke.

The importance of this review lies in its potential to inform clinical practice and guide future research. We aim to identify the optimal ONSD measurement in predicting malignant MCA infarct. By synthesizing existing data, we can better understand the role of ONSD in stroke care. Furthermore, this review will highlight the practical implications of ONSD measurements, offering insights into how this tool can be integrated into stroke management protocols to improve patient outcomes. Given the lack of recent systematic reviews on this topic, this study is both timely and necessary, providing an updated evidence base for clinicians and researchers alike.

## Review

Research design

This study was a systematic review and meta-analysis conducted in accordance with the Preferred Reporting Items for Systematic Reviews and Meta-Analyses (PRISMA) 2022 guidelines.

Search strategies and study selection

**Figure 1:**
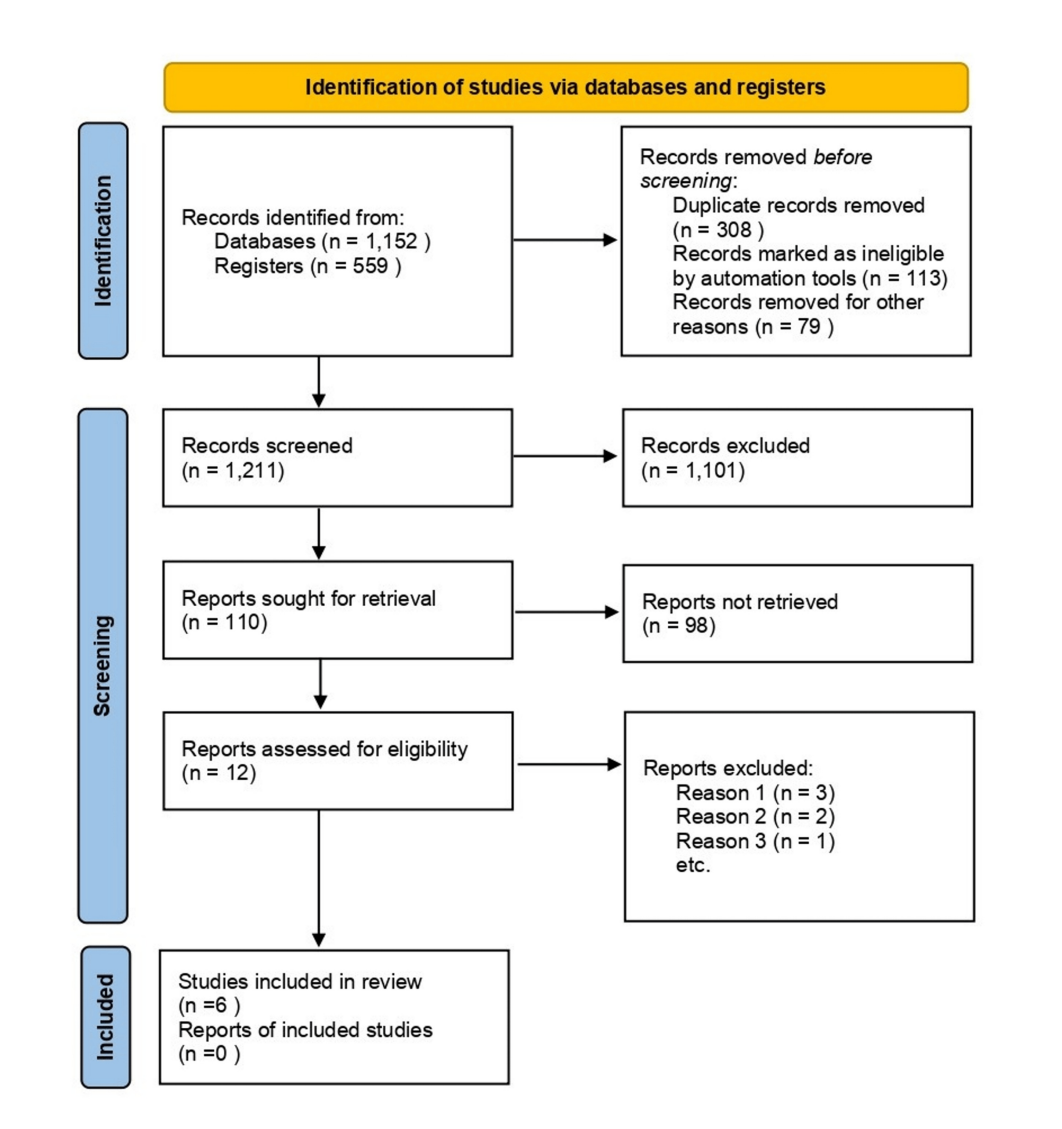
Preferred Reporting Items for Systematic Reviews and Meta-Analysis (PRISMA) guidelines 2022

Figure 1 shows the summary of steps in the identification of studies via databases and registers. PubMed, Cochrane, and Google Scholar databases were used to look for available studies. The search was conducted in September 2024. The search terms used were the following: malignant supratentorial infarct OR malignant middle cerebral artery infarction OR space-occupying brain edema OR large hemispheric infarction OR ischemic stroke OR cerebral infarction OR brain edema OR intracranial pressure OR stroke progression AND optic nerve sheath diameter OR ONSD measurement OR ultrasonography OR ultrasound OR noninvasive monitoring OR intracranial pressure monitoring OR ICP monitoring AND diagnostic accuracy OR prognostic value OR clinical outcomes OR mortality OR functional disability OR feasibility OR reliability OR cost-effectiveness. All of the studies retrieved were in English and were appraised for bias by the researcher using the Newcastle-Ottawa Scale (NOS) for observational studies [8]. The full text of the studies was reviewed and tabulated using MS Excel (Microsoft Corporation, Redmond, Washington, United States). The review protocol was not registered in any systematic review database and is acknowledged by the authors as a limitation of the study.

Eligibility criteria

Inclusion Criteria

This review included studies that focused on adult patients, aged 18 years and older, who were diagnosed with malignant supratentorial infarcts, specifically MCA infarctions, confirmed through neuroimaging such as CT or MRI. 

Studies that involved mixed populations, including stroke patients with other neurological conditions, were included if they provided data specific to malignant supratentorial infarcts. Additionally, studies that utilized ONSD measurement via ultrasonography as a noninvasive method for monitoring ICP were considered. Research employing standard ICP monitoring techniques, such as invasive methods (e.g., intraventricular catheters) or advanced imaging (e.g., MRI or CT), was also included, even in the absence of a comparator group, as long as ONSD measurements were reported in the context of malignant supratentorial infarcts.

Eligible studies reported primary outcomes such as the accuracy of ONSD in detecting elevated ICP, its correlation with clinical outcomes, including mortality and functional disability, and its predictive value for malignant progression. Secondary outcomes included the feasibility of ONSD measurement, interobserver reliability, and cost-effectiveness. The review included observational study designs such as prospective and retrospective cohort studies, case-control studies, and cross-sectional studies. Studies conducted in various clinical settings, including intensive care units, emergency departments, and stroke units, were considered as long as relevant outcomes were reported.

Furthermore, only studies published between January 2000 and December 2023 were included to ensure relevance to current clinical practices. Due to resource constraints, only English-language publications were considered. Both peer-reviewed journal articles and preprints were eligible for inclusion. Lastly, studies were required to have a minimum follow-up period of 24 hours to allow for the assessment of the short-term predictive value of ONSD measurements.

Exclusion Criteria

Studies were excluded from the review if they did not measure or report outcomes specifically related to ONSD measurements in the context of malignant supratentorial infarcts. Research that focused solely on other neurological conditions, such as traumatic brain injury or subarachnoid hemorrhage, without providing data relevant to stroke patients, was also excluded. Additionally, studies with inadequate methodological quality determined using the NOS for observational studies were not considered. Case reports, editorials, and narrative reviews were excluded due to their limited scientific rigor. Conference abstracts and unpublished manuscripts were also excluded.

Data collection and quality assessment

The researcher extracted the data from the full articles and stored it in Mendeley Reference Manager. Using the inclusion/exclusion criteria, articles during the search were retrieved. Disagreements were resolved by consultation with a third reviewer.

In this study, a meta-analysis was conducted to pool estimates of mean ONSD measurement, sensitivity, specificity, positive predictive value (PPV), negative predictive value (NPV), and accuracy using the metamean() and metaprop() functions from the meta R package. For all six studies, the sample size, mean ONSD measurement, and corresponding standard deviation were extracted. The ONSD measurements used for the meta-analysis of means were those taken immediately after admission to the emergency department or within the same day of hospital admission following stroke onset, as reported in the respective studies. Event counts were derived from reported true positives (TP), false positives (FP), true negatives (TN), and false negatives (FN). Studies with missing or nonreported values for specific variables (e.g., detection rate or diagnostic accuracy metrics) were excluded from the corresponding meta-analyses but were included in analyses where adequate data were available. A random-effects model was used to account for potential variability across study populations, imaging modalities, and measurement protocols. Heterogeneity was assessed using the I² statistic, tau², and Cochran’s Q test. Forest plots were generated using a RevMan-style layout, including 95% confidence intervals for each study and the pooled estimate.

To estimate an optimal ONSD threshold, we compared the diagnostic performance of cutoff values reported across studies where such data are available. Youden's Index (sensitivity + specificity − 1) was calculated for each study to evaluate the diagnostic efficiency of the reported cutoffs. A higher Youden's Index indicates better discriminatory ability. The cutoff value with the highest Youden's Index was considered to provide the best trade-off between sensitivity and specificity.

Visualization of Youden’s Index across different cutoff values was performed using line plots generated with ggplot2. This allowed for the identification of threshold regions with consistent diagnostic performance.

Study characteristics

In the six studies that were included in the study for meta-analysis (Table 1), three were retrospective observational studies and three were prospective observational studies. A total of 480 participants, including 89 malignant infarct cases, 211 nonmalignant infarct cases, and 180 healthy subjects. These six studies utilized different imaging modalities, such as ocular ultrasound, computed tomography (CT), and MRI, to evaluate ONSD and its utility in identifying malignant progression in stroke patients, particularly those with large MCA infarcts.

**Table 1:** Characteristic summary of the six included studies ONSD: optic nerve sheath diameter; MCA: malignant cerebral artery; ETD: eyeball transverse diameter; MRI: magnetic resonance imaging; ICP: intracranial pressure

Study	Study design	Sample size	Population	Remarks
Patel et al. (2021) [9]	Prospective observational study	86	Stroke patients admitted to ED/ICU	Elevations in optic nerve sheath diameter were associated with increased in hospital mortality and poor functional outcome at 6 months
Kozaci et al. (2019) [10]	Prospective observational study	200	Stroke patients and healthy controls using ocular ultrasound	Optic nerve sheath diameter increases earlier than computerized tomography and diffusion-weighted imaging alterations
Lochner et al. (2020) [11]	Prospective observational study	29	Stroke patients with healthy controls	ONSD measurement might be accurate for the noninvasive detection of increased ICP and for the recognition of patients being likely to develop malignant MCA infarction
Legros et al. (2021) [12]	Retrospective observational study	88	Malignant stroke patients with matched healthy controls	Optic nerve and perioptic sheath diameter in the first MRI can predict the risk of developing a malignant MCA infarct
Guo et al. (2020) [13]	Retrospective observational study	91	Ischemic stroke patients with middle cerebral artery (MCA) infarcts	Increased ONSD or ONSD/ETD ratio increases the odds of malignant progression and may be used as an indicator for emergent therapeutic interventions
Lee et al. (2020) [14]	Retrospective observational study	58	Stroke patients with large infarcts; some developed malignant cerebral edema	The rate of ONSD/ETD changes compared to baseline at D1 CT can be a predictor of late malignant progression

Three studies, Patel et al. [9], Kozaci et al. [10], and Lochner et al. [11] were prospective in design. The other three studies, Legros et al. [12], Guo et al. [13], and Lee et al. [14] were retrospective observational in design.

Kozaci et al. [10] was the largest study included with 200 subjects, which included 100 ischemic stroke patients and 100 healthy controls. ONSD was assessed using ocular ultrasound and stratified by CT and DWI findings. Guo et al. [13] have 91 subjects with MCA infarction and used nonenhanced CT to determine ONSD and ONSD/ETD ratio. Lee et al. [14] have 58 subjects, Lochner et al. [11] have 29 MCA infarct subjects and 14 healthy controls, Legros et al. [12] have included 22 subjects who required decompressive hemicraniectomy and compared them to 66 matched controls using MRI, and Patel et al. [9] have 86 subjects. The six studies required subjects to have confirmed ischemic strokes, while common exclusion factors included ocular pathology such as glaucoma and cataract.

Risk of bias assessment

The risk of bias in Table 2 for each study included was assessed using the NOS, a tool for assessing nonrandomized or observational studies. NOS has three domains, namely, selection, comparability, and outcome.

**Table 2:** Summary of risk of bias in each study assessed using Newcastle-Ottawa Scale (NOS) Selection: (1) representativeness of the exposed cohort, (2) selection of the nonexposed cohort, (3) ascertainment of exposure, (4) demonstration that the outcome was not present at the start. Comparability: (1) Comparability of cohorts on the basis of design or analysis. Outcome: (1) Assessment of outcome. (2) Was the follow-up long enough for outcomes to occur? (3) Adequacy of follow-up

Study	Selection (max of 4 stars)				Comparability (max of 2 stars)	Outcome (max of 3 stars)			Total score	Risk for bias
	1	2	3	4	1	1	2	3		
Patel et al. (2021) [9]	*	*	*	*	**	*	*	*	9	Low risk
Kozaci et al. (2019) [10]	*	*	*	*	*	*	*	*	8	Low risk
Lochner et al. (2020) [11]	*	*	*	*	**	*	*	*	9	Low risk
Legros et al. (2021) [12]	*	*	*	*	**	*	*		8	Low risk
Guo et al. (2022) [13]	*	*	*	*	*	*	*	*	8	Low risk
Lee et al. (2020) [14]	*	*	*	*	**	*	*		8	Low risk

Two studies, Patel et al. [9] and Lochner et al. [11], scored perfect (9 out of 9). This shows strong methodological rigor, including clear inclusion criteria, complete follow-up, validated ONSD measurement methods, and appropriate adjustment for confounders. Four studies, Guo et al. [13], Kozaci et al. [10], Lee et al. [14], and Legros et al. [12], each scored 8 out of 9 stars. They lose one star due to either limited adjustment for confounding variables or incomplete follow-up reporting.

All studies clearly defined participant selection criteria, employed standardized and validated imaging methods (ultrasound, CT, or MRI) to assess ONSD, and applied consistent procedures across groups. Most were adjusted for or matched key clinical and demographic factors. All six of the studies assessed have a low risk of bias.

Results

Detection Rate

The meta-analysis of detection rate included four independent studies, encompassing a total of 170 individuals identified as having either malignant MCA infarction or progression or acute ischemic stroke. Among these, 154 individuals were reported as having been correctly detected, resulting in a high overall event rate. The pooled detection rate, calculated using both the common-effect and random-effects models, was 0.8901 with a 95% confidence interval of 0.8288 to 0.9313, reflecting a consistently high performance across studies. The identical estimates from both models indicate strong agreement and suggest minimal variation in detection rates between studies.

**Figure 2:**
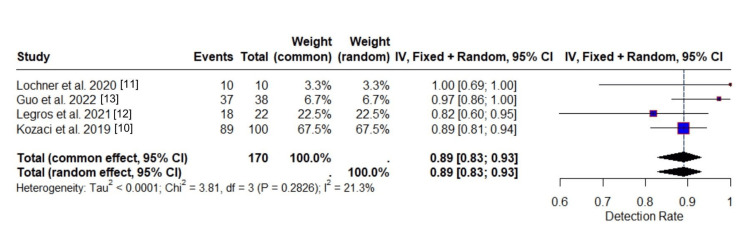
Forest plot of detection rate across studies

Figure 2 illustrates individual study estimates alongside the overall pooled detection rate. Kozaci et al. [10] contributed the greatest weight to the analysis (67.5%), with a detection rate of 0.89, closely matching the pooled estimate. Legros et al. [12] reported a slightly lower detection rate of 0.82 (22.5% weight), while Guo et al. [13] had a higher rate of 0.97 (6.7% weight). Lochner et al. [11] demonstrated perfect detection (1.00), though its influence on the pooled estimate was limited due to its smaller sample size (3.3% weight). The pooled estimate is represented by the diamond at the bottom of the plot and reiterates the high overall detection rate of 0.8901 (95% CI: 0.8288 to 0.9313).

Heterogeneity assessment showed minimal between-study variance (τ² < 0.0001), with an I² value of 21.3%, indicating that the small presence of heterogeneity might not be important. Cochran’s Q-test (Q = 3.81, df = 3, p = 0.2826) did not reach statistical significance, supporting the assumption of homogeneity across studies. Collectively, these findings suggest that the detection rates reported across the included studies are consistent and reliable.

Mean ONSD Measurement

Six independent studies were included for the meta-analysis of means to estimate the pooled mean of ONSD measurements. The analysis included a total of 314 observations. Using a random-effects model, the pooled mean ONSD was 5.50 mm with a 95% confidence interval of 5.24 to 5.76 mm, reflecting the average measurement across studies.

**Figure 3:**
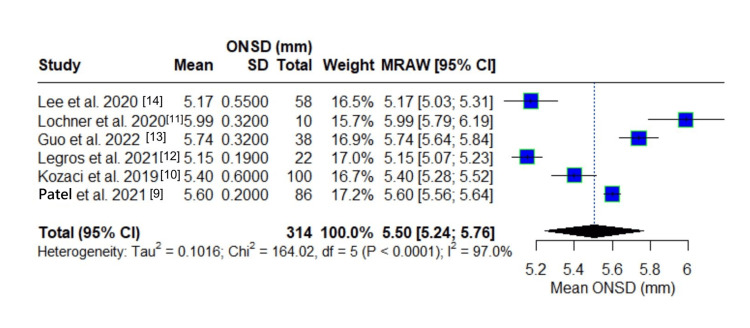
Forest plot of mean ONSD measurement ONSD: optic nerve sheath diameter

Figure 3 shows the individual study results. Lochner et al. [11] reported the highest mean ONSD of 5.99 mm, approximately 0.49 mm above the pooled estimate. This represents the greatest positive deviation from the pooled mean, with confidence intervals that minimally overlap with the pooled estimate. Conversely, Legros et al. [12] reported the lowest mean ONSD of 5.15 mm, which falls 0.35 mm below the pooled mean and shows minimal overlap with the pooled confidence interval. Kozaci et al. [10] and Guo et al. [13] reported intermediate values of 5.40 mm and 5.74 mm, respectively, deviating -0.10 mm and +0.24 mm from the pooled estimate. Lee et al. [14] with a mean of 5.17 mm showed substantial negative deviation (-0.33 mm) from the pooled estimate, while Patel et al. [9] reported a mean ONSD of 5.60 mm, closely approximating the pooled estimate (+0.10 mm).

Assessment of heterogeneity indicated substantial variability in mean estimates between studies. The between-study variance (τ²) was estimated at 0.1016. The I² statistic was 97.0%, translating to considerable heterogeneity and suggesting that a large proportion of the observed variance was due to true differences between studies rather than sampling error. This is further supported by the Cochran’s Q statistic (Q = 164.02, df = 5, p < 0.0001), which indicates statistically significant heterogeneity.

Diagnostic Performance

The meta-analysis for sensitivity, specificity, PPV, NPV, and accuracy was performed across the same four independent studies. Two other studies were excluded due to missing data.

The results for the sensitivity diagnostic reveal a pooled proportion, calculated using both the common-effect model and the random-effects model, of 0.8901 (95% CI: 0.8288 to 0.9313), reflecting a high occurrence of the correct identification of subjects with the characteristic of interest across all studies. This result is consistent with the findings from the detection rate analysis, where a high rate of detection was similarly observed across studies, reinforcing the reliability and uniformity of the outcome. A sensitivity of 89% thus correlates with a high likelihood of identifying patients with malignant infarction in triage settings or identifying progression of the infarct in the ICU setting.

**Figure 4:**
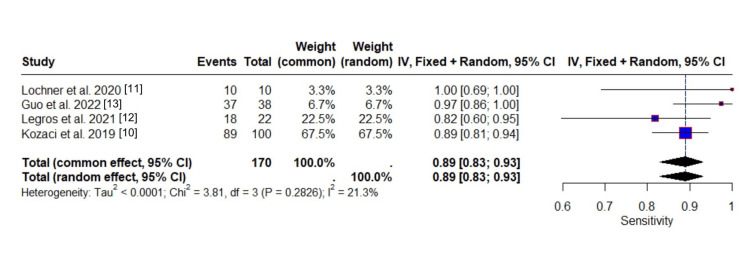
Forest plot showing the sensitivity of included studies

Figure 4 visually supports this consistency, showing overlapping confidence intervals for each study and identical diamonds for the pooled estimates from both the common-effect and random-effects models, further emphasizing the stability of the results.

Heterogeneity was assessed in the same way as in the detection rate analysis. The between-study variance (τ²) was negligible (<0.0001), and the I² statistic was 21.3%, indicating low heterogeneity. These findings mirror those from the detection rate analysis, suggesting minimal variability across the studies. Furthermore, the Cochran’s Q-test (Q = 3.81, df = 3, p = 0.2826) showed no significant heterogeneity, reinforcing the conclusion that the studies are largely consistent in their findings.

The evaluation of the specificity of ONSD in identifying patients without malignant MCA infarction or malignant progression involved 238 observations represented individuals classified as not having malignant progression, while 204 events reflected those correctly identified as such, i.e., true negatives. The common-effect model estimated a pooled specificity of 0.8294 (95% CI: 0.7686 to 0.8768), whereas the random-effects model yielded a slightly higher estimate of 0.8663 (95% CI: 0.7126 to 0.9443). The discrepancy between the two models suggests notable variability in effect sizes across studies, with the random-effects model incorporating greater uncertainty and allowing for between-study differences. The high specificity for ONSD ensures that individuals with malignant MCA infarction are accurately identified among patients with acute ischemic stroke.

**Figure 5:**
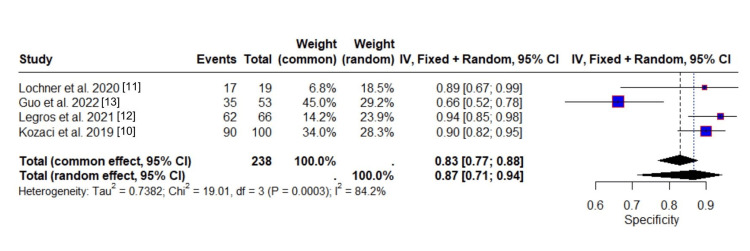
Forest plot of specificity results

Figure 5 illustrates how individual studies contribute to this heterogeneity. Legros et al. [12] and Kozaci et al. [10] reported the highest specificity values (0.94 and 0.90, respectively), clustering near the upper end of the scale. Lochner et al. [11] demonstrated similarly high specificity (0.89) but with broader confidence intervals, reflecting a smaller sample size. In contrast, Guo et al. [13] reported a notably lower specificity of 0.66, diverging from other studies and showing minimal confidence interval overlap, further underscoring heterogeneity. From a clinical perspective, high specificity is critical for minimizing false positives, that is, avoiding misclassifying patients as having malignant progression when they do not.

Quantitative assessments confirm substantial heterogeneity across studies. The between-study variance (τ²) was estimated at 0.7382. The I² statistic was 84.2%, indicating a substantial amount of heterogeneity. This is supported by Cochran’s Q-test (Q = 19.01, df = 3, p = 0.0003), which detected statistically significant heterogeneity. However, when Guo et al. (2022) was excluded from the analysis, the I² statistic dropped to 0%, suggesting that the observed heterogeneity was largely driven by this study. This indicates that, without Guo et al.'s results, the remaining studies show very little variation in specificity estimates.

The evaluation for the PPV of ONSD measurements involves a total of 154 events, representing individuals correctly identified as having malignant progression or MCA infarction (i.e., true positives). The random-effects model estimated a pooled PPV of 0.8137, indicating that, on average, approximately 81% of patients with a positive test result truly had the condition of interest. The common-effect model produced a closely comparable estimate of 0.7993.

**Figure 6:**
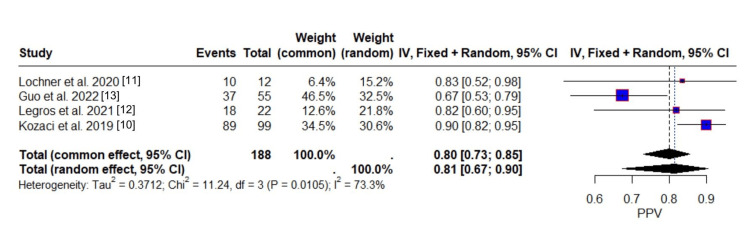
Forest plot of positive predictive value across studies

The forest plot of PPV in Figure 6 highlights considerable variability across studies. Individual PPV estimates ranged from 0.67 [13] to 0.90 [10], with Guo et al. [13] reporting notably lower predictive accuracy than the other studies. This study also exerted substantial influence on the pooled estimate, accounting for 46.5% of the weight under the common-effect model and 32.5% under the random-effects model. The vertical reference line around 0.80 contextualizes the spread of study estimates, with some falling above and others falling below this threshold.

Assessment of heterogeneity revealed substantial between-study variability. The I² statistic was 73.3% indicating substantial heterogeneity, and the between-study variance (τ²) was estimated at 0.3712, indicating moderate to high inconsistency in effect sizes. The Cochran’s Q-test was statistically significant (Q = 11.24, df = 3, p = 0.0105), further confirming that observed variation across studies exceeded what would be expected by chance alone.

Importantly, a sensitivity analysis excluding Guo et al. [13], the study with the lowest PPV, resulted in an I² of 0%, suggesting that this single study was the primary driver of heterogeneity. This finding underscores the influence of individual study characteristics, such as population demographics or test implementation protocols, on diagnostic accuracy outcomes.

The PPV 81 % of that ONSD implies its advantage in triage settings and early recognition in the ICU. A high PPV is crucial, as it reflects the test’s ability to correctly identify individuals who truly have the condition when a positive result is obtained. Thus, the generally high pooled PPV across studies supports the utility of ONSD as a reliable diagnostic indicator, while also highlighting the importance of consistent testing protocols to reduce variability.

The NPV evaluation included a total of 204 true negative events, representing individuals correctly identified as not having malignant progression or MCA infarction. The pooled NPV estimated under the random-effects model was 0.9257, indicating that approximately 93% of individuals with a negative test result were accurately classified as not having the condition. The common-effect model yielded a closely aligned estimate of 0.9170.

**Figure 7:**
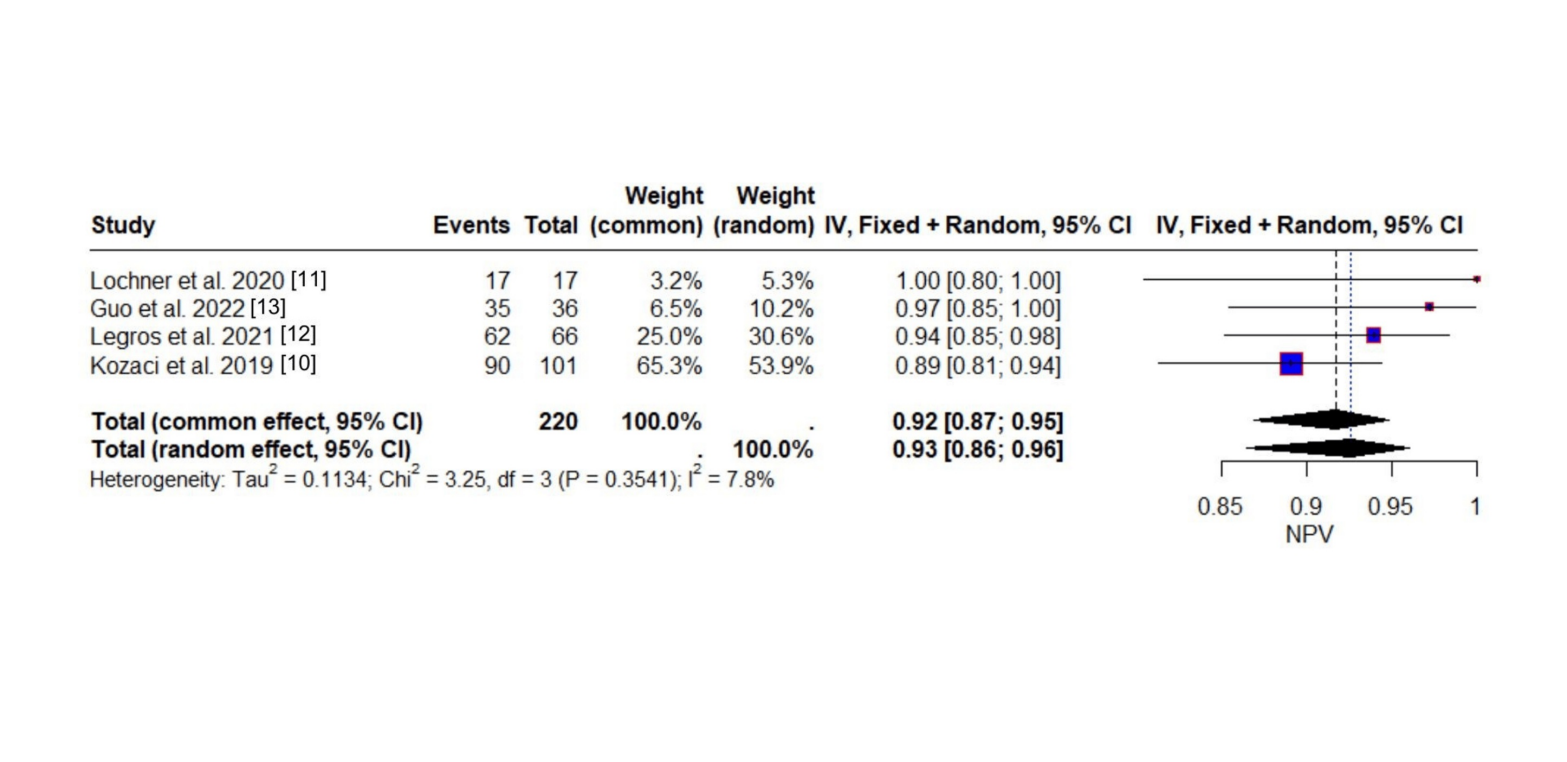
Forest plot of negative predictive value across studies

The forest plot in Figure 7 reveals that NPV estimates were consistently high across studies, ranging from 0.89 [10] to 1.00 [11]. All studies reported NPVs of ≥0.89, with minimal variation between them, and all studies have overlapping confidence intervals. Kozaci et al. [10] contributed the greatest statistical weight (65.3% under the common-effect model; 53.9% under the random-effects model), while Lochner et al., despite reporting perfect specificity, contributed the least (3.2% and 5.3%, respectively), likely due to a smaller sample size. The reference line near 0.90 visually reinforces the overall high accuracy of negative test results, with study estimates tightly clustered around or above this threshold.

The heterogeneity assessment reveals a high level of consistency across studies. The I² statistic was only 7.8%, and the between-study variance (τ²) was estimated at 0.1134, both indicating minimal heterogeneity. Cochran’s Q-test was nonsignificant (Q = 3.25, df = 3, p = 0.3541), which means the small differences in NPV across the studies are likely due to random chance. This suggests that the studies are consistent with each other, and the high NPV is a reliable finding. The NPV of 93% supports the utility of ONSD measurement as a safe and effective screening tool for excluding malignant progression, reinforcing its role in early triage and decision-making pathways.

Assessing diagnostic accuracy involves evaluating the number of subjects correctly identified as having malignant progression or MCA (true positives), as well as those correctly identified as nonprogressions or without malignant MCA (true negatives). The pooled proportion of diagnostic accuracy, based on the random-effects model, was estimated at 0.8782 (95% CI: 0.8073 to 0.9254), indicating that, on average, approximately 88% of cases were correctly classified across the included studies. The common-effect model yielded a similar estimate of 0.8706 (95% CI: 0.8330 to 0.9007), suggesting a generally high level of diagnostic accuracy. This high accuracy is particularly relevant because it highlights the effectiveness of the diagnostic test in correctly identifying both those with and without the condition, which is crucial in clinical decision-making.

**Figure 8:**
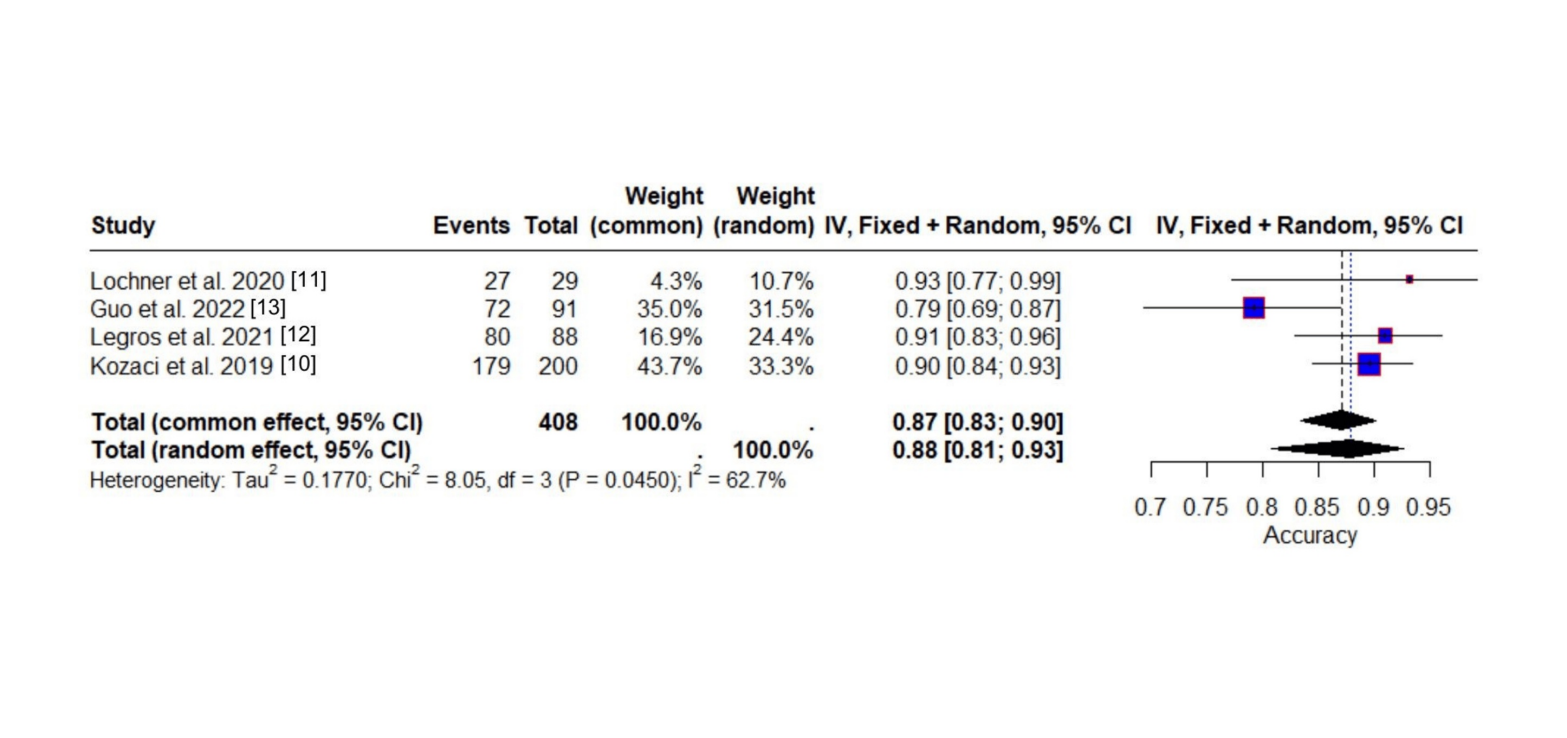
Forest plot of diagnostic accuracy of ONSD thresholds ONSD: optic nerve sheath diameter

The individual study accuracies, shown in Figure 8, range from 0.79 [13] to 0.93 [11], with three studies demonstrating accuracy ≥0.90. Guo et al. stand out with an accuracy of 0.79, notably lower than the other studies. Kozaci et al. [10] contributed the greatest weight in the common-effect model (43.7%) due to its large sample size, followed by Guo et al. [13] (35.0%). In the random-effects model, the weights were more evenly distributed, with (33.3%) [10], (31.5%) [13], and 24.4% [12] all making substantial contributions. The pooled estimates are visualized as overlapping diamonds at the bottom of the plot, and the reference line around 0.87 helps illustrate how Guo et al.'s lower accuracy falls notably below the pooled estimate, while the other studies cluster above it. The confidence intervals for all studies, except Guo, overlap with each other, confirming that Guo et al. is the primary source of heterogeneity in this analysis. When Guo et al. is excluded from the analysis, the I² statistic drops to 0%, indicating no remaining heterogeneity, which suggests that Guo's study is the main contributor to the variability observed across studies.

Heterogeneity assessment revealed moderate variability among studies, with an I² statistic of 62.7%, indicating substantial heterogeneity. The estimated between-study variance (τ²) was 0.1770, and the Cochran’s Q test was statistically significant (Q = 8.05, df = 3, p = 0.0450), suggesting that the differences between studies were larger than expected by chance alone and should be considered.

Optimal Cutoff Value for ONSD Measurement

Table 3 summarizes the optimal cutoff values for optic nerve sheath diameter (ONSD) identified in four independent studies, alongside their corresponding diagnostic performance metrics, sensitivity, specificity, and Youden’s Index. The cutoff values ranged from 4.70 mm to 5.60 mm, with the highest Youden’s Index observed in Lochner et al. at a cutoff of 5.60 mm, indicating excellent diagnostic discrimination (sensitivity = 1.0000, specificity = 0.8947, Youden’s Index = 0.8947). Other studies reported cutoffs of 5.03 mm, 5.20 mm, and 4.70 mm, with corresponding Youden's Index values of 0.7574, 0.6341, and 0.7900, respectively.

**Table 3:** Youden’s Index across studies ONSD: optic nerve sheath diameter

Study	Cut-off ONSD measurement (mm)	Sensitivity	Specificity	Youden’s Index
Kozaci et al. (2019) [10]	4.7	0.89	0.9	0.79
Lochner et al. (2020) [11]	5.6	1	0.8947	0.8947
Legros et al. (2021) [12]	5.03	0.8181	0.9393	0.7574
Guo et al. (2022) [13]	5.2	0.9737	0.6604	0.6341

**Figure 9:**
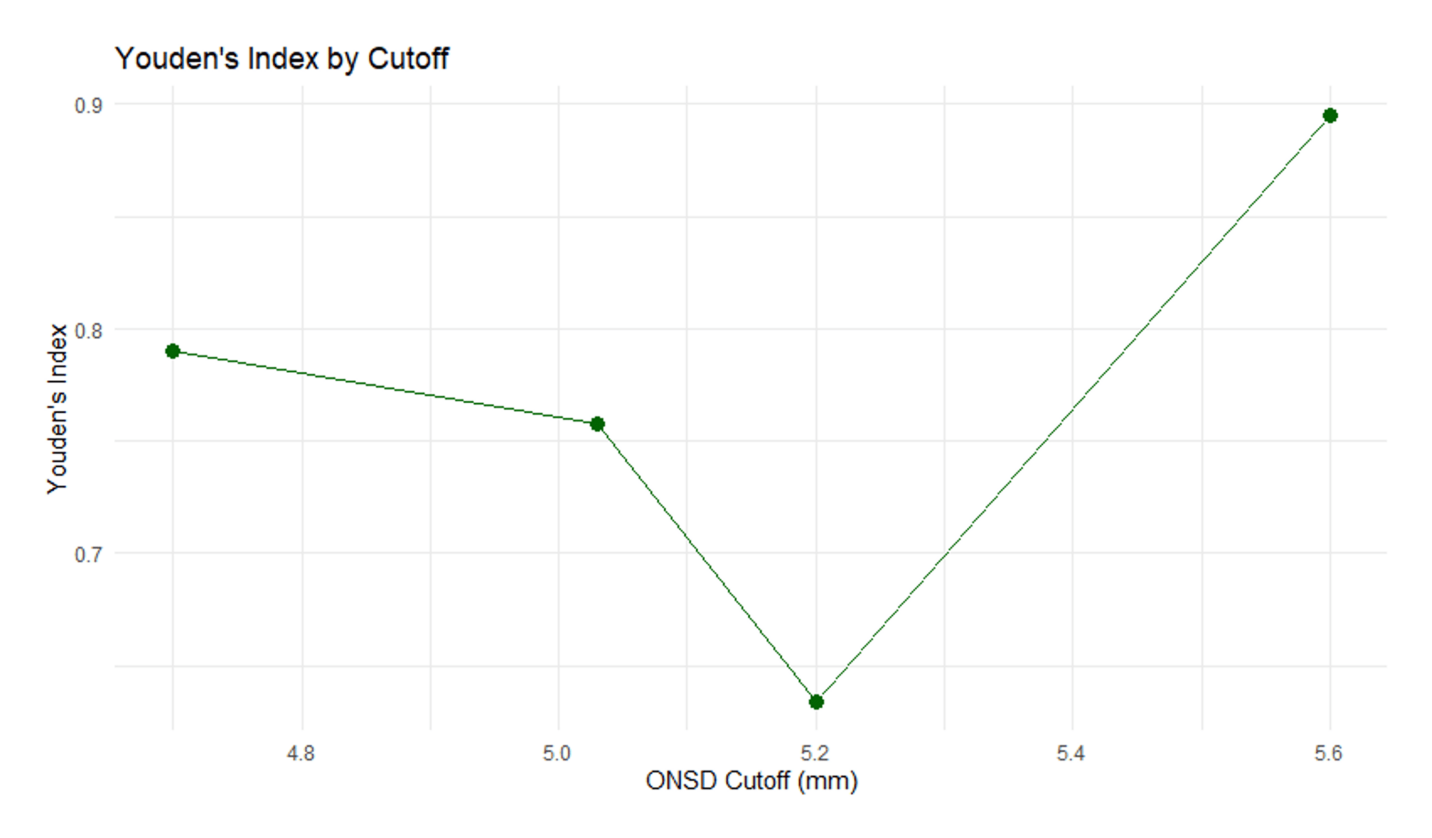
Youden’s Index curve across ONSD threshold ONSD: optic nerve sheath diameter Source: [9-14]

Figure 9 displays Youden's Index plotted against different ONSD cutoff values measured in millimeters. The graph shows a U-shaped relationship with Youden's Index. This indicates that among the tested cutoff values, an ONSD measurement of 5.6 mm provides the optimal balance of sensitivity and specificity for diagnostic purposes, as reflected by the highest Youden's Index value.

Importantly, all reported cutoff values fall within the 95% confidence interval (5.24 mm to 5.76 mm) of the pooled mean ONSD derived from the random-effects meta-analysis. While that mean estimate showed high heterogeneity (I² = 97.0%), the fact that these cutoffs lie within its confidence range suggests a general consistency in the diagnostic threshold across studies, despite between-study variation in mean ONSD values.

These findings support the notion that a clinically relevant ONSD threshold likely falls to 5.6 mm, reinforcing the potential utility of this measurement in identifying patients with elevated ICP or related acute neurologic conditions.

Discussion

The optic nerve sheath is continuous with the subarachnoid space of the brain, allowing cerebrospinal fluid (CSF) pressure fluctuations to be transmitted directly to the optic nerve sheath [13,15]. Therefore, any rise in ICP, such as that caused by malignant cerebral edema following infarction, results in measurable sheath dilation, making ONSD a reliable surrogate marker for ICP elevation. In this study, the high detection rate (89.01%), with minimal heterogeneity (I² = 21.3%) findings, shows a strong diagnostic consistency of ONSD across different studies. The mean ONSD across studies was 5.50 mm (95% CI: 5.24-5.76 mm), although substantial heterogeneity (I² = 97.0%) was observed.

A high ONSD sensitivity at 89.01% shows that most cases of malignant infarction were correctly identified. Specificity of 82.94% to 86.63% indicates a relatively few false positives, consistent with prior reports that ONSD enlargement is specific to elevated ICP [16]. A PPV of 81.37% and an NPV of 92.57% suggest that ONSD is particularly effective at ruling out malignant infarction if measurements are normal. The study of Guo et al. contributed disproportionately to heterogeneity in specificity and PPV, likely due to different patient populations or technical factors, as seen in prior literature variability regarding ICP and ONSD thresholds [13].

The optimal cutoff value for ONSD to predict malignant infarction was identified as 5.6 mm, with the highest diagnostic yield (Youden's Index = 0.8947). This finding aligns with the pathophysiologic concept that significant ICP elevations cause expansion of the optic nerve sheath, which can be reliably detected around 3 mm posterior to the globe, the site of maximal distensibility [17-18].

The meta-analysis of the study shows that the early detection of raised ICP via ONSD measurement can help detect the clinical deterioration even before overt neurologic signs manifest, enabling timely interventions like decompressive surgery, which improves outcomes when performed early [17]. Systematic reviews and meta-analyses for specific disease entities are mostly limited to traumatic brain injury. This study is unique in that we have focused on acute ischemic stroke. Publication bias is minimal because of the small number of studies available. Future research would include multicenter studies and machine learning, and measurements of ONSD. Comparative studies can be done over other noninvasive methods for ICP monitoring in stroke patients, as baseline data for ONSD measurement is being established [19].

However, even though the results are promising, several limitations must be acknowledged. First, operator dependency in ultrasound-based ONSD measurement can affect consistency, particularly in emergency or high-stress clinical settings. Variability in measurement protocols (e.g., monocular vs. binocular measurements and interobserver variability) also contributes to the heterogeneity observed across studies. In terms of variability, other intrinsic factors that contributed are evolving imaging techniques and variable patient demographics.

## Conclusions

ONSD measurement is a highly sensitive and specific noninvasive technique for identifying malignant supratentorial infarcts. An ONSD threshold of 5.6 mm offers optimal diagnostic performance. Grounded in solid anatomic and physiologic principles and supported by consistent results across different modalities, ONSD measurement should be considered a valuable adjunct in the recognition of acute ischemic stroke at risk of malignant progression, especially where access to advanced imaging is limited or in the stroke unit, where the measurement can be done at bedside easily. In triage scenarios, ONSD measurement may provide immediate identification and timely management and referrals for malignant MCA infarcts.
